# Synergistic Action of Biosynthesized Silver Nanoparticles and Culture Supernatant of *Bacillus amyloliquefacience* against the Soft Rot Pathogen *Dickeya dadantii*

**DOI:** 10.3390/plants12091817

**Published:** 2023-04-28

**Authors:** Afsana Hossain, Jinyan Luo, Md. Arshad Ali, Rongyao Chai, Muhammad Shahid, Temoor Ahmed, Mohamed M. Hassan, Roqayah H. Kadi, Qianli An, Bin Li, Yanli Wang

**Affiliations:** 1 State Key Laboratory for Quality and Safety of Agro-Products, Institute of Plant Protection and Microbiology, Zhejiang Academy of Agricultural Sciences, Hangzhou 310021, China; afsana_07@yahoo.com (A.H.); rychai@sina.com (R.C.); 2 Institute of Biotechnology, Zhejiang University, Hangzhou 310058, China; alibau201@gmail.com (M.A.A.);; 3 Department of Plant Pathology, Bangabandhu Sheikh Mujibur Rahman Agricultural University, Gazipur 1706, Bangladesh; 4 Department of Plant Quarantine, Shanghai Extension and Service Center of Agriculture Technology, Shanghai 201103, China; 5 Department of Bioinformatics and Biotechnology, Government College University, Faisalabad 38000, Pakistan; 6 Department of Biology, College of Science, Taif University, Taif 21944, Saudi Arabia; 7 Department of Biology, Faculty of Science, University of Jeddah, Jeddah 21959, Saudi Arabia

**Keywords:** silver nanoparticles, synergistic action, *Dickeya dadantii*, sweet potato

## Abstract

Nanomaterials are increasingly being used for crop growth, especially as a new paradigm for plant disease management. Among the other nanomaterials, silver nanoparticles (AgNPs) draw a great deal of attention because of their unique features and multiple usages. Rapid expansion in nanotechnology and utilization of AgNPs in a large range of areas resulted in the substantial release of these nanoparticles into the soil and water environment, causing concern for the safety of ecosystems and phytosanitary. In an attempt to find an effective control measure for sweet potato soft rot disease, the pathogen *Dickeya dadantii* was exposed to AgNPs, the cell-free culture supernatant (CFCS) of *Bacillus amyloliquefaciens* alone, and both in combination. AgNPs were synthesized using CFCS of *Bacillus amyloliquefaciens* strain A3. The green synthesized AgNPs exhibited a characteristic surface plasmon resonance peak at 410–420 nm. Electron microscopy and X-ray diffraction spectroscopy determined the nanocrystalline nature and 20–100 nm diameters of AgNPs. Release of metal Ag^+^ ion from biosynthesized AgNPs increases with time. AgNPs and CFCS of *B. amyloliquefaciens* alone exhibited antibacterial activity against the growth, biofilm formation, swimming motility, and virulence of strain A3. The antibacterial activities elevated with the elevation in AgNPs and CFCS concentration. Similar antibacterial activities against *D. dadantii* were obtained with AgNPs at 50 µg·mL^−1^, 50% CFCS alone, and the combination of AgNPs at 12 µg·mL^−1^ and 12% CFCS of *B. amyloliquefaciens*. In planta experiments indicated that all the treatments reduced *D. dadantii* infection and increased plant growth. These findings suggest that AgNPs along with CFCS of *B. amyloliquefaciens* can be applied to minimize this bacterial disease by controlling pathogen-contaminated sweet potato tuber with minimum Ag nano-pollutant in the environment.

## 1. Introduction

The application of nanoscale-size materials possessing no less than one dimension in the 1 to 100 nanometers range is a newly emerging area in nanoscience and technology. The nanomaterials display exceptional physical, chemical, and electrical properties for their extreme surface-to-volume ratio [1]. The extensive use of nanoparticles raises concern about their environmental impact, but the impact of NPs on ecological processes and organisms remains largely unknown. Enormous production and application of NPs release nano-size materials into the environment during the production process as well as using, and these NPs have an unknown consequence on ecosystem structure and functioning when they enter aquatic and terrestrial environmental processes [1,2].

Among the diverse types of metal nanoparticles, silver nanoparticles gained boundless attention for application in multiple disciplines, especially in the agricultural field, because of their exclusive properties such as chemical stability, good conductivity, catalytic, and, most importantly, antimicrobial activities [3,4,5]. Silver nanoparticles exhibit antibacterial activity using different mechanisms, although these mechanisms have yet to be fully elucidated. Many studies state that AgNPs can disrupt the cell wall; increase intercellular ROS production, oxidative stress, and photocatalytic activity; and inhibit cell division and replication by binding to cell walls, the S-H group of proteins, and the N7 atom of guanin [6,7]. Green synthesis of silver nanoparticles attained worldwide attention due to their minimum use of resources and energy as well as for their biocompatibility and environmental safety [8,9]. The growing use of AgNPs inevitably increases the chance of releasing toxic Ag^+^ into the environment. Research showed that AgNPs at any level of concentration caused oxidative stress and serious inhibition of photosynthesis, which ultimately retarded the growth of plants and exhibited higher toxicity [10,11].

Gram-positive bacteria belonging to the genera *Bacillus* are found in nature and inhibit other pathogenic microorganisms present in the rhizosphere and seeds. They are an effective and eco-friendly method for the biological control of a large number of plant fungal and bacterial diseases [12,13]. *Bacillus* spp. are the best candidates for producing effective biopesticides because of their spore-forming ability and resistance to dryness [14]. *Bacillus* can produce a vast array of biologically active molecules, such as non-ribosomally synthesized peptides and lipopeptides, polyketides, bacteriocins, and siderophores, that are important and can potentially inhibit phytopathogenic bacteria [14,15]. Studies have shown that lipopeptides present in the culture filtrate of *Bacillus* have biocontrol capacity against fungus and bacteria [15,16,17].

The control of bacterial disease mostly relies on chemical pesticides. The broad-spectrum use of chemical pesticides has resulted in the development of multi-drug resistant bacteria, which is a main threat to controlling bacterial infection. Limiting the chemical pesticide application rate in agriculture is one of the main objectives for current agricultural production. This can be achieved in diverse ways, either by introducing new or more effective alternatives, such as nanoparticles, or by the combination of nanoparticles with other effective alternatives, such as plant extracts or culture filtrates of antagonistic fungi and bacteria. The use of AgNPs combined with culture filtrate may become a popular alternative as this method reduces the risk of hazardous Ag^+^. The combination of two or more tested materials can reduce the amount of Ag^+^ needed to control pathogens and the negative consequences of AgNPs on human health and nature [1,18]. Therefore, the control of bacterial pathogens using biosynthesized nanoparticles and *Bacillus* can be an effective alternative to chemical pesticides [7,19].

Gram-negative bacteria from the genera of *Pectobacterium* and *Dickeya* [20] are broad-host-range pathogens, which can attack many crop and ornamental plants [21]. They are causative agents of destructive soft rot disease for various vegetable and ornamental plants due to the production of pectinase, which can degrade pectin in the primary walls and middle lamella of plant cell walls, resulting in plant tissue maceration [22,23]. Over the last two decades, foot rot in rice [24,25], stalk rot in maize [26], black leg and soft rot in potato [27,28,29], stem and root rot in sweet potato [30,31], and soft rot and sheath rot in banana [32,33] caused by some aggressive species of *Dickeya* have hampered staple food security. Few crop varieties are resistant to *Dickeya* and *Pectobacterium*, while the control of soft rot in agricultural fields is highly dependent on effective antibiotic use, which will not be feasible in the future because of the threat of environmental pollution and antibiotic-resistant human and animal pathogens. However, the increased consumer demand for chemical-free foods has motivated researchers to find promising prevention and control strategies that can serve as alternatives to chemical pesticides. Antagonistic *Bacillus*, *Paenibacillus* biosynthesized AgNPs, ZnONPs, and TiO_2_NPs can potentially inhibit *Dickeya dadantii* causing stem and root rot on sweet potato [34,35,36].

Recently, *Dickeya dadantii* soft rot was found in most sweet potato growing areas in China, indicating a need for an efficient strategy to control this pathogen. The objective of this study was to determine the potentiality of the combination of CFCS and AgNPs against *D. dadantii*.

## 2. Results and Discussion

In our previous study [37], the in vivo and in vitro antibacterial activity of 21 different *Bacillus* strains were screened against *D. dadantii*. Among all *Bacillus* strains, *B. amyloliquefaciens* A3 showed significantly higher antibacterial activity against *D. dadantii* compared to other *Bacillus* strains. Furthermore, silver nanoparticles were successfully synthesized using the cell-free culture supernatant of *B. amyloliquefaciens* A3 with the incubation of silver nitrate [35,38].

### 2.1. Characterization of Synthesized AgNPs with B. amyloliquefaciens CFCS

**Figure 1:**
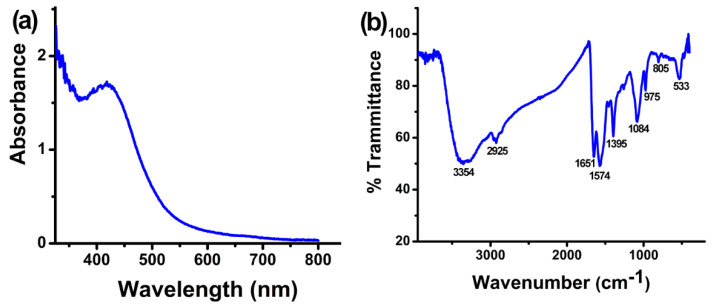
Characterization of cell-free culture supernatant (CFCS) of *Bacillus amyloliquefaciens* mediated silver nanoparticles (**a**) Ultra violet-visible spectrum of newly synthesized AgNPs solution. Synthesized AgNPs exhibit a clear surface plasmon resonance peak at 410–420 nm. (**b**) Fourier transform infrared spectrum displaying functional group accountable for the AgNPs synthesis and their stabilization.

AgNPs were synthesized by incubating the CFCS of *B. amyloliquefaciens* and AgNO_3_ (3 mM). After 48 h, the mixture changed to a dark brown color from light yellow, which indicated the reduction of Ag^+^ into Ag^0^ in AgNO_3_ solutions. UV-Vis spectrometry confirmed the existence of synthesized AgNPs in the solution [39], which showed a characteristic surface plasmon resonance peak at 410–420 nm (Figure 1a). Previous studies showed that free electrons of silver nanoparticles exhibited surface plasmon resonance at 410 to 450 nm [38,40,41,42].

FTIR spectrometry revealed the functional groups in the CFCS of *B. amyloliquefaciens* A3 that cause the reduction of Ag^+^, stabilization, and AgNPs capping. The FTIR spectrum of the synthesized AgNPs exhibited absorption peaks at 3354, 2925, 1651, 1574, 1395, 1084, 975, 805, and 533 cm^−1^ (Figure 1b). The peak at 3354 cm^−1^ is for N–H stretching vibration; the peak at 2925 cm^−1^ corresponds to C–H stretching vibration and N–H stretching of the amide group; the peak at 1651 cm^−1^ corresponds to –C=O and C=C stretching vibrations; the band at 1574 cm^−1^ is attributed to C=C stretching vibration; the peak at 1395 cm^−1^ is attributed to CH_3_ or C–H bending; the peak at 975 cm^−1^ corresponds to C=C bending and C–H vibration; and the peaks at 1084 cm^−1^, 805 cm^−1^, and 533 cm^−1^ are for C=O stretching, C–H bending, and C–C skeleton vibration, respectively. The existence of these functional groups confirmed the presence of protein in the CFCS of *B. amyloliquefaciens* and also indicated that these groups act as reducing and stabilizing agents in the synthesis process of AgNPs [43,44]. Earlier reports showed that free amine groups as well as cysteine residue in proteins can combine with nanoparticles and stabilize the AgNPs [45].

**Figure 2:**
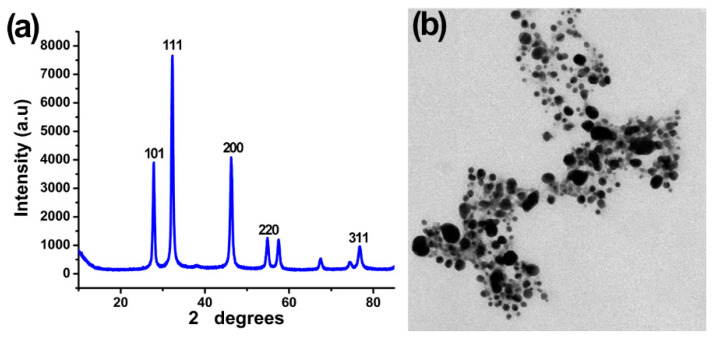
Characterization of cell-free culture supernatant (CFCS) of *Bacillus amyloliquefaciens* mediated silver nanoparticles (**a**) X-ray diffraction spectrum demonstrating the nanosize and crystalline nature of the AgNPs (**b**) TEM displaying AgNPs with a diameter of 20–100 nm and spherical form.

The X-ray diffraction patterns revealed the nanocrystalline nature of synthesized AgNPs. Based on the Bragg reflection peak at 2θ, the values of 27.86, 32.27, 46.22, 54.86, and 76.74 correspond to planes (101), (111), (200), (220), and (311), respectively (Figure 2a). The four reflection peaks are correlated with the crystallographic nature of the centering face and the cubic nature of AgNPs, which was confirmed with a comparison to the powder diffraction card of the Joint Committee on Powder Diffraction Standard File No. JCPDS 04-0783. The average particle diameter of AgNPs is 50.3 nm, which was calculated using the Debye–Scherrer equation:

*D* = *Kλ*/(*β Cosθ*),

where *D* represents the median crystalline nanoparticles size, *K* (0.94) represents the Scherrer constant, *λ* (0.15406 nm) represents the wavelength of X-ray, *β* represents the full breadth at half largest of the X-ray diffraction peak, and *θ* represents the Bragg angle.

**Figure 3:**
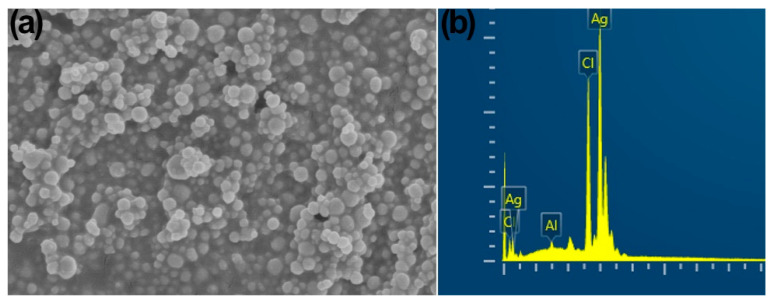
Characterization of cell-free culture supernatant (CFCS) of *Bacillus amyloliquefaciens* mediated silver nanoparticles (**a**) SEM displaying AgNPs with a diameter of 20–100 nm and spherical form. (**b**) Energy dispersive spectrum displaying the prevalence of Ag element in the AgNPs.

Transmission (TEM) electron microscopy and scanning (SEM) electron microscopy were used to confirm the AgNP sizes that were synthesized from the CFCS of *B. amyloliquefaciens*. The TEM images (Figure 2b) showed that most of the AgNPs had uniform size, were mono-dispersed, and about 20–100 nm in diameter with spherical shape and an average mean size of 48.5 nm, which were confirmed with the SEM images (Figure 3a). These results are consistent with previous studies [35,43].

Energy dispersive spectroscopy further confirmed the dominance of metal silver (Ag^0^) in the synthesized AgNPs. The typical absorption peak of the Ag element was exhibited by AgNPs at 3 KeV (Figure 3b), which confirmed that nano-sized silver was in a pure form, as earlier reports have shown [35,38,41,46,47,48]. Spectroscopy showed 82.32 wt% Ag^0^ along with 17.07 and 0.61 wt% of Cl and Al, respectively.

### 2.2. Metal Ag Release

**Figure 4:**
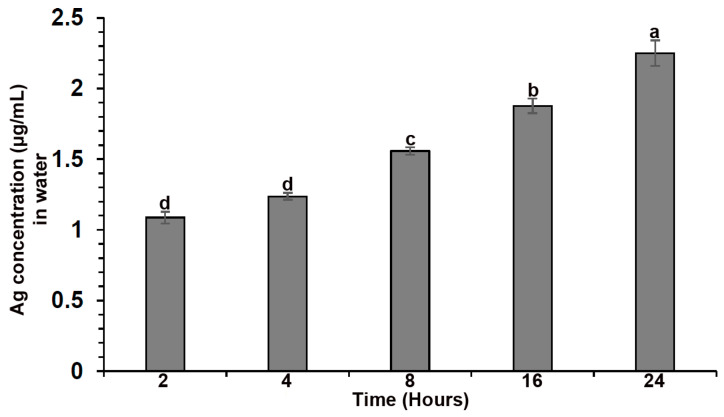
Release of Ag^+^ ions during different time intervals. Different lowercase letters on the bars are represented the significantly different at *p* ≤ 0.05.

The metal release data indicated that biosynthesized AgNPs significantly release Ag^+^ ions in ddH_2_0 during every time interval (Figure 4). After 8 h, a quick initial release of Ag^+^ ions from the biosynthesized AgNPs was observed, which then attained a persistent value. Biosynthesized AgNPs released the most Ag^+^ (2.25) after 24 h. Previous studies found that there is a correlation between the suppression of disease and the ion release profile [49]. Moreover, components of different media have a specific function in releasing metal ions from nanoparticles.

### 2.3. Antibacterial Activity of the Combination of CFCS and AgNPs against D. dadantii

AgNPs (50 µL), CFCS (50%), and the combination of AgNPs (12 µL) and CFCS (12%) exhibit a similar extended of inhibition of growth and swimming motility against *D. dadantii* strain A3 and tissue maceration in sweet potato. The inhibition of in vivo tissue maceration using tuber slices was in general agreement with the result of in vitro bacterial growth, biofilm formation, and swimming motility.

AgNPs synthesized with *B. amyloliquefaciens* at different concentrations (12, 24, and 50 µL) and the CFCS of *B. amyloliquefaciens* at different percentage (12, 24, and 50%) can significantly inhibit *D. dadantii* growth in liquid broth. Inhibition increases with the increase in concentration of AgNPs or percentage of CFCS (Table 1 and Table 2). The highest inhibition of *D. dadantii* growth was found using 50 µL AgNPs and 50% CFCS. The combination of AgNPs (12 µL) and CFCS (12%) can significantly inhibit *D. dadantii* growth at a similar extent as 50 µL AgNPs and 50% CFCS (Table 3).

After 24 h of incubation at 30 °C without shaking, *D. dadantii* formed biofilm on the surface of the polystyrene plate. The combination of AgNPs (12 µL) and CFCS (12%) significantly inhibited (72%) the formation of biofilm, whereas AgNPs (50 µL) and CFCS (50%) alone showed a similar extent of inhibition (Table 4).

*Dickeya dadantii* grew and showed swimming ability in semi-solid media with 0.3% (*w*/*v*) agar and formed a 35 mm diameter halo after 48 h of incubation at 30 °C (Table 4). The combination of AgNPs (12 µL) and CFCS (12%) significantly inhibited (68%) the halo diameter, whereas AgNPs (50 µL) and CFCS (50%) alone showed a similar extent of inhibition.

In sweet potato tubers, *D. dadantii* created a maceration zone by degrading plant cell walls in the sweet potato slice. One day after inoculation, the maceration zone was about 32 mm (Table 4). The combination of AgNPs (12 µL) and CFCS (12%) significantly inhibited (65%) tissue maceration, whereas AgNPs (50 µL) and CFCS (50%) alone showed a similar extent of inhibition.

**Figure 5:**
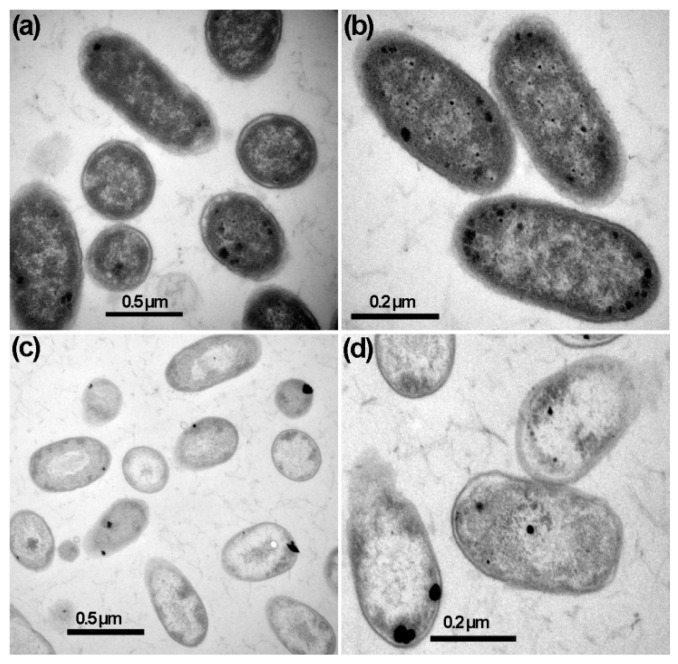
Transmission electron micrograph (TEM) images showing *Dickeya dadantii* cells without treatment (**a**,**b**) and those treated with the combination of silver nanoparticles (12 µL) and cell-free culture supernatant (12%) (**c**,**d**). Intact cell walls and dense cytoplasm characterized the control cells without treatment (**a**,**b**). Disintegration and clearing of the cytoplasm were evident in cells after 4 h of treatment (**c**,**d**).

TEM also revealed the morphological changes in *D. dadantii* cells after treatment with the combination of AgNPs (12 µL) and CFCS (12%). After 4 h of treatment, most *D. dadantii* cells were dead, as indicated by distorted cells with disintegrated cytoplasm and cell walls (Figure 5c,d). On the other hand, *D. dadantii* cells grown without treatment had intact cell walls with dense cytoplasm filled in the cell (Figure 5a,b).

During the in planta experiment, *D. dadantii* was inoculated into sweet potato seed tubers, and due to the infection, the tubers became rotten. *D. dadantii* was then recovered from the rotten inoculated tubers. Seed tubers treated with water germinated and became small seedlings 21 days post-inoculation. On the other hand, seed tubers treated with AgNPs (50 µg·mL^−1^) from the CFCS of *B. amyloliquefaciens* A3, the CFCS of *B. amyloliquefaciens* A3 (50%), and the combination of AgNPs (12 µg·mL^−1^) and CFCS (12%) germinated and became seedlings with a greater height than the seedlings grown from the water-treated seed tubers (Table 5). The fresh weight and dry weight of seedlings grown from sweet potato seed tubers treated with AgNPs (50 µg·mL^−1^) from the CFCS of *B. amyloliquefaciens* A3, the CFCS of *B. amyloliquefaciens* A3 (50%), and the combination of AgNPs (12 µg·mL^−1^) and CFCS (12%) were higher than the fresh and dry weight of the seedlings grown from water-treated seed tubers (Table 5).

AgNPs may transport Ag^+^ more efficiently to the bacterial membrane and cytoplasm [50]. Ag^+^ release from the 20–80 nm silver nanoparticle and subsequent penetration to the bacterial cell membrane might be the primary cause for the antibacterial action of AgNPs [50,51,52,53]. The CFCS of *B. amyloliquefaciens*-derived AgNPs were about 20–100 nm in width and inhibited the growth, biofilm formation, and swimming motility of *D. dadantii* through attachment to surfaces of bacterial cells, which may release toxic Ag^+^ and damage the cell membrane. After penetration into the *D. dadantii* cells, AgNPs may generate oxidative stress and interact with the sulfur present in the membrane proteins and the phosphorous remaining in the DNA to disrupt the respiratory chain and cell division, which lead to cell death [53,54]. The antimicrobial actions of AgNPs are mainly due to the release of Ag^+^, and the release of Ag^+^ even in very low concentrations can account for the biological response [50].

On the other hand, *Bacillus* spp. are known to be active against a large range of phytopathogenic bacteria through competition with phytopathogens and antibiosis by producing antibiotics and metabolites. As a biocontrol agent, the main attribute of *Bacillus* spp. is their various secondary metabolites and capability to produce a wide range of structurally various antimicrobial substances [15]. In comparison with other isolates, 8% of the whole genome of plant-associated *B. amyloliquefaciens* strain FZB42 is involved in the production of secondary metabolites such as bacteriocins, antimicrobial peptides and lipopeptides, polyketides, and siderophore with microbial activities [55,56]. A higher concentration of surfactin was observed in the CFCS of *B. amyloliquefaciens* strain A3 among the effective strain against *D. dadantii* [37]. Surfactins belonging to lipopeptides with strong antibacterial activities [14,57] may play a key role in the inhibition of growth and biofilm formation of *D. dadantii*. Previous studies showed that silver nanoparticles synthesized with the incubation of the CFCS of *Bacillus amyloliquefaciens* strain A3 also exhibited antibacterial activities [35,38]. Selection of *Bacillus amyloliquefaciens* strain A3 can be used for the production of CFCS and the synthesis of AgNPs, which avoids the screening of bacteria and can be used as a reference strain for the synthesis of AgNPs.

In conclusion, we provide a simple, environmentally safe, and economically viable control strategy to combat the bacterial pathogen *Dickeya dadantii* using a combination of AgNPs and CFCS. *Dickeya dadantii*-infected sweet potato can be treated with the combination of AgNPs (12 µg) and CFCS (12%) to produce a healthy seed crop. This can minimize the labor required to produce a large amount of CFCS and reduce the chance of releasing Ag^+^ from AgNPs. The combination of AgNPs and CFCS acts as a *Dickeya dadantii* inhibitor and growth promoter for the plant. The promising potency of the combination of AgNPs and cell-free culture supernatant can draw immense research interest in the production of green-synthesized nano pesticides.

## 3. Materials and Methods

### 3.1. Bacteria

The pathogenic strain CZ1501 of *D. dadantii* was isolated from infected sweet potato stems cultivated in Hangzhou of Zhejiang province, China, and the biocontrol strain *B. amyloliquefaciens* strain A3 isolated from rice seeds [58] were used in this study. Both bacteria were grown in nutrient agar or broth medium consisting of 10 g of tryptone, 2.5 g of glucose, 5 g of NaCl, and 3 g of beef extract with or without 15 g of agar per liter, respectively, at pH 7.0.

### 3.2. Preparation of CFCS and Synthesis of AgNPs

*Bacillus amyloliquefaciens* strain A3 was cultured in NA broth at 30 °C for 48 h at 200 rpm. CFCS was prepared with centrifugation at 27,200× *g* for 15 min and then two times filtration with a 0.22 µm millipore filter. A lack of bacterial presence was confirmed by culturing 100 µL CFCS in nutrient agar for 24 h at 30 °C.

To the biosynthesized AgNPs, 35 mL CFCS of *B. amyloliquefaciens* A3 was mixed with 115 mL of a freshly prepared aqueous solution of 3 mM AgNO_3_ (Cat. No. 10018461; Sinopharm, Shanghai, China) in a 250 mL Erlenmeyer flask and kept under shaking at 200 rpm and 30 °C for 48 h in dark conditions. Then, 35 mL of nutrient broth and 115 mL of 3 mM AgNO_3_ were added together and used as the control. AgNPs synthesis was observed when the solution changed from a light yellow to dark brown color. After adding 2 mL of the dark brown solution with 2 mL of Milli-Q water, the UV-Visible spectra were observed at 1 nm resolution in the range of 200–750 nm using a Simadzu UV-2550 spectrometer (Shimadzu, Kyoto, Japan). AgNPs were obtained from the dark brown solution with centrifugation at 27,200× *g* for 10 min followed by washing two times with Milli-Q water, after which they were freeze-dried with Alpha 1-2 LD plus (GmbH, Marburg, Germany). The freeze-dried AgNPs were weighed, and a stock solution of 50 mg.mL^−1^ was prepared by dissolving AgNPs with Milli-Q water.

### 3.3. Characterization of AgNPs Synthesized with CFCS with B. amyloliquefaciens

The functional groups for the biosynthesis and stabilization of AgNPs were confirmed using Fourier transforms infrared (FTIR) spectroscopy. In detail, 1 mg of freeze-dried AgNP powder was mixed with 300 mg KBr, and thin pellets were made using a hydraulic pellet press. FTIR spectrum was documented with an AVATAR 370 FTIR spectrometer (Thermo Nicolet, Madison, WI, USA) in the range between 500 and 4000 cm^−1^ with a 4 cm^−1^ resolution. FTIR spectra were interpreted using the table of Bruker infrared (Bruker Optics Inc., Billerica, MA, USA) and the absorption table of Libre Texts infrared spectroscopy (https://chem.libretexts.prg/) accessed on 8 June 2018.

To observe the AgNP size and morphology, SEM and TEM were used. One drop of the AgNPs solution was applied onto the carbon-coated copper grid and then dried under a mercury lamp. The SU8010 field emission scanning electron microscope (Hitachi, Tokyo, Japan) and a JEM-1230 transmission electron microscope (JEOL, Tokyo, Japan) were used to observe the grid carried in AgNPs. An X-Max N energy dispersive spectrometer (Oxford Instruments, Oxford, UK) was used to detect the silver element in AgNPs at 20 keV.

The X-ray diffraction pattern was used to understand the crystallographic structure of AgNPs. A coated film of freeze-dried AgNP powder was kept on the glass slide and analyzed using a D8 Advance Diffractometer (Bruker, Karlsruhe, Germany) maintaining an operational condition of 40 kV and 40 mA with a Cu–Kα radiation ranging from 20° to 80° at the 2θ angle.

### 3.4. Experiments on Ag Release

The metal Ag release from the biosynthesized AgNPs was calculated in sterile ddH_2_0 according to the previously described method [59] with a few modifications. In brief, suspensions were prepared using 25 µg·mL^−1^ AgNPs in ddH_2_0 with three replications, and the pH was adjusted to 7. The suspensions were incubated at 26 ± 2 °C under continuous shaking at 150 rpm. After 2, 4, 8, 16, and 24 h time intervals, 1 mL aliquot was taken from each suspension and centrifuged at 13,000× *g* to discard NPs. The acquired supernatants were digested by applying HClO_4_:HNO_3_ (1:3), and the Ag content was analyzed using inductively coupled plasma-mass spectrometry (ICP-MS; Optima 8000DV, Pekin-Elmer, Waltham, MA, USA). The quality of the investigative data was ensured by running a standard suspension containing 100 µg·mL^−1^ continually in each of the 5 samples.

### 3.5. Antibacterial Assay

*D. dadantii* CZ1501 was grown in nutrient broth at 30 °C with shaking at 200 rpm to the mid-exponential phase, and the concentration of bacterial cells was adjusted to about 5 × 10^8^ CFU∙mL^−1^ before use.

Antibacterial activities of AgNPs synthesized with the CFCS of *B. amyloliquefaciens* strain A3, the CFCS of *B. amyloliquefaciens* strain A3, and the combination of AgNPs with CFCS were determined in the nutrient broth. Briefly, 5 mL of nutrient broth was inoculated with 100 µL *D. dadantii* suspensions and used as the control. AgNPs from *B. amyloliquefaciens* strain A3 were adjusted to concentrations of 12, 25, and 50 µg·mL^−1^ by adding nutrient broth. Furthermore, 100 µL *D. dadantii* suspensions were inoculated into 5 mL of nutrient broth containing AgNPs. *D. dadantii* suspensions (100 µL), NA broth (2.4 mL), the CFCS of *B. amyloliquefaciens* A3 (0.6 mL or 1.25 mL or 2.5 mL), and sterile distilled water (1.9 mL or 1.25 mL or 0 mL) to a total volume of 5 mL were added into a glass tube to obtain final concentrations of 12, 25, and 50% CFCS. *D. dadantii* suspensions (100 µL), NA broth (2.4 mL), the CFCS of A3 (0.6 mL or 1.25 mL or 2.5 mL), and sterile distilled water (1.9 mL or 1.25 mL or 0 mL) were mixed, and AgNPs were added with nutrient broth consisting of CFCS to obtain concentrations of 12, 25, and 50 µg·mL^−1^. The samples were then incubated at 30 °C and 200 rpm for 24 h. After that, the optical density of the samples was measured at 600 nm (OD_600_) using a SpectraMax spectrophotometer (Molecular Devices, Sunnyvale, CA, USA). This experiment was repeated three times with three replications of each treatment.

The effect of 50 µg·mL^−1^ synthesized AgNPs from *B. amyloliquefaciens* strain A3, 50% of the CFCS of *B. amyloliquefaciens* strain A3, and the combination of AgNPs (12 µg·mL^−1^) and CFCS (12%) on the swimming motility of *D*. *dadantii* strain CZ1501 was determined with semi-solid nutrient agar [0.3% (*w*/*v*)]. A plate without AgNPs and CFCS served as the control. AgNPs were added to semisolid nutrient agar [0.3%, *w*/*v*)] to a concentration of 50 µg·mL^−1^. Equal amounts of the CFCF of *B. amyloliquefaciens* strain A3 and semisolid nutrient agar [0.6% (*w*/*v*)] were mixed to obtain a 50% CFCS concentration. AgNPs and CFCS were added to the semi-solid nutrient agar to obtain a final concentration of 12 µL AgNPs and 12% of CFCS. In the center of each swimming plate, a suspension (5 µL) of *D. dadantii* was spotted and incubated for 48 h at 30 °C. The diameter of *D. dadantii* colonies was measured. The experiment was repeated three times with three replicates of each treatment.

The inhibition of biofilm formed by *D*. *dadantii* strain CZ1501 using AgNPs (50 µg·mL^−1^) from *B. amyloliquefaciens* strain A3, CFCS of *B. amyloliquefaciens* strain A3 (50%), the combination of AgNPs (12 µg·mL^−1^) and CFCS (12%) was determined using the method of crystal violet staining, which was quantified by measuring the absorbance at 590 nm (OD_590_) using a 96-well microtiter plate. AgNPs were added with nutrient broth to obtain a concentration of 50 µg·mL^−1^. AgNPs were added with nutrient broth consisting of 12% CFCS to a concentration of 12 µg∙mL^−1^. An amount of 100 µL *D. dadantii* suspensions was mixed with 100 µL of the CFCS of *B. amyloliquefaciens* A3 or nutrient broth composed of 50 µg·mL^−1^ AgNPs (100 µL) or nutrient broth containing 12 µg∙mL^−1^ AgNPs along with 12% CFCS (100 µL). The mixtures were independently added to the wells of a 96-well microplate. Furthermore, a 100 µL of *D. dadantii* suspension added with 100 µL of distilled water was used as the control, whereas 100 µL of nutrient broth with 100 µL of distilled water was used as the blank. The plates were then incubated without shaking at 30 °C for 24 h. The liquid from each well was discarded and gently washed three times using distilled water followed by 1 h of air drying. In addition, 200 µL crystal violet (1%, *w*/*v*) was used to stain the bacterial cells that attached to the wells. A thorough washing was performed after incubation for 30 min at room temperature using distilled water. The crystal violet dye in the bacterial cells was dissolved by adding 33% (*v*/*v*) of 200 µL acetic acid in each well. The OD value at 590 nm was measured using SpectraMax spectrophotometer (Molecular Device, Sunnyvale, CA, USA). The experiment was repeated three times with six replicates of each treatment.

The effect of AgNPs (50 µg·mL^−1^) from *B. amyloliquefaciens* strain A3, the CFCS of *B. amyloliquefaciens* strain A3 (50%), and the combination of AgNPs (12 µg·mL^−1^) and CFCS (12%) on in vivo antagonistic actions against *D. dadantii* was assayed with tuber slices of sweet potato. After surface sterilization with 70% (*v*/*v*) ethanol, the sweet potato tubers were rinsed with sterile distilled water, and 10 mm thick slices were prepared. The tuber slices were kept in a solution containing 50 µg·mL^−1^ AgNPs or 50% CFCS or 12 µg∙mL^−1^ AgNPs along with 12% CFCS for 1 h, and after that, they were air-dried for 1 h in Petri dishes. Then, 5 µL of *D. dadantii* cell suspension was spotted by puncturing at the center of the slice. The slices were incubated at 30 °C for 24 h. The diameter of the maceration zones created by *D. dadantii* around the punctures was measured. The experiment was repeated three times with three replicates of each treatment.

### 3.6. Transmission Electron Microscopy

The combined effect of AgNPs and CFCS on the structure of *D. dadantii* cells was determined using TEM. *D. dadantii* suspension alone or *D. dadantii* suspension containing a combination of AgNPs (12 µg·mL^−1^) and CFCS of *B. amyloliquefaciens* A3 (12%) were incubated at rotary shaking at 200 rpm and 30 °C for 4 h. The sample for TEM analysis was prepared followed by harvesting *D. dadantii* cells as described in our previous work [35], and observations were made using the JEM-1230 transmission electron microscope.

### 3.7. In Planta Assay

An in planta experiment was assayed to evaluate the efficacy of AgNPs (50 µg·mL^−1^) derived from the CFCS of *B. amyloliquefaciens* strain A3, the CFCS of *B. amyloliquefaciens* strain A3 (50%), and the combination of AgNPs (12 µg·mL^−1^) and CFCS (12%) for the growth promotion of sweet potato seedlings and the suppression of *D. dadantii* according to the method of [37] with slight modifications. In brief, after surface sterilization with 70% (*v*/*v*) ethanol, the sweet potato tubers were rinsed with sterile distilled water and immersed in *D. dadantii* suspension (1 × 10^7^ CFU mL^−1^) or in sterile distilled water for 4 h. The seed tubers were air dried for 12 h and then immersed in sterile distilled water or AgNPs (50 µg·mL^−1^) from *B. amyloliquefaciens* strain A3, *B. amyloliquefaciens* strain A3-originated CFCS (50%), or the combination of AgNPs (12 µg·mL^−1^) and CFCS (12%) for 4 h. After treatment, all seed tubers were kept on three-layered moistened and sterilized filter papers in Petri dishes of 15 cm diameter. A single seed tuber was placed in a petri dish and was kept in a growth chamber for 21 days, maintaining 28 °C temperature, 80% humidity, and a 12/12-h light/dark photoperiod. After 21 days of incubation, seedling height was measured. The experiment was repeated three times with six replications of each treatment.

### 3.8. Statistical Analysis

Experimental data were analyzed using SPSS software version 16 (SPSS, Chicago, IL, USA) at a *p* < 0.05 level of significance. All data are presented as the mean of at least three values with their standard error for each independent experiment.

## Figures and Tables

**Table 1:** Growth of *D. dadantii* in liquid nutrient broth containing CFCS (12, 25, or 50%) corresponded to an optical density at 600 nm (OD_600_).

Treatment	Value at OD_600_
*D. dadantii*	1.01 ± 0.02 d
*D. dadantii* + 12%	0.59 ± 0.03 c
*D. dadantii* + 25%	0.34 ± 0.03 b
*D. dadantii* + 50%	0.13 ± 0.02 a

Different letters within the same column indicate a statistically significant difference (*p* < 0.05).

**Table 2:** Growth of *D. dadantii* in liquid nutrient broth containing AgNPs (12, 25, or 50 µg·mL^−1^) corresponding to an optical density at 600 nm.

Treatment	Value at OD_600_
*D. dadantii*	1.02 ± 0.03 d
*D. dadantii* + AgNPs (12 µg)	0.44 ± 0.03 c
*D. dadantii* + AgNPs (25 µg)	0.33 ± 0.02 b
*D. dadantii* + AgNPs (50 µg)	0.12 ± 0.02 a

Different letters within the same column indicate a statistically significant difference (*p* < 0.05).

**Table 3:** Growth of *D. dadantii* in liquid nutrient broth containing CFCS (50%), AgNPs (50 µg·mL^−1^), and the combination of CFCS (12%) and AgNPs (12 µg·mL^−1^) corresponding to an optical density at 600 nm.

Treatment	Value at OD_600_
*D. dadantii*	1.04 ± 0.03 b
*D. dadantii* + CFCS (50%)	0.13 ± 0.02 a
*D. dadantii* + AgNPs (50 µg)	0.12 ± 0.02 a
*D. dadantii* + CFCS (12%) + AgNPs (12 µg)	0.11 ± 0.02 a

Different letters within the same column indicate a statistically significant difference (*p* < 0.05).

**Table 4:** Biofilm formation, swimming motility of *D. dadantii,* and the diameter of macerated tissue generated by *D. dadantii* in sweet potato tuber slices with CFCS (50%), AgNPs (50 µg·mL^−1^), and the combination of CFCS (12%) and AgNPs (12 µg·mL^−1^).

Treatment	Value at OD570	Colony Diameter (mm)	Tissue Maceration (mm)
*D. dadantii*	0.187 ± 0.04 b	30.70 ± 1.00 b	34.95 ± 0.79 b
*D. dadantii* + CFCS (50%)	0.062 ± 0.04 a	13.52 ± 0.84 a	13.20 ± 1.25 a
*D. dadantii* + AgNPs (50 µg)	0.055 ± 0.04 a	12.38 ± 0.83 a	12.13 ± 0.98 a
*D. dadantii* + CFCS (12%) + AgNPs (12 µg)	0.051 ± 0.04 a	10.03 ± 0.89 a	11.10 ± 0.94 a

Different letters within the same column indicate a statistically significant difference (*p* < 0.05).

**Table 5:** Seedling height, fresh and dry weight of seedlings raised from sweet potato seed tubers treated with water, *D. dadantii*, *D. dadantii* with the CFCS of *B. amyloliquefaciens* A3 (50%), AgNPs (50 µg·mL^−1^) from the CFCS of *B. amyloliquefaciens* A3, or the combination of AgNPs (12 µg·mL^−1^) and CFCS (12%).

Treatment	Seedling Height (cm)	Fresh Weight (gm)	Dry Weight (gm)
Water	2.5 ± 0.09 b	0.29 ± 0.04 b	0.04 ± 0.01 b
*D. dadantii*	0.0 ± 0.00 c	0.00 ± 0.00 c	0.00 ± 0.00 c
*D. dadantii* + CFCS (50%)	6.3 ± 0.22 a	0.73 ± 0.04 a	0.13 ± 0.02 a
*D. dadantii* + AgNPs (50 µg)	6.2 ± 0.27 a	0.71 ± 0.03 a	0.10 ± 0.01 a
*D. dadantii* + CFCS (12%) + AgNPs (12 µg)	6.6 ± 0.27 a	0.76 ± 0.04 a	0.14 ± 0.02 a

Different letters within the same column indicate a statistically significant difference (*p* < 0.05).

## Data Availability

The data is contained within the manuscript.
